# *Drosophila* as a toolkit to tackle cancer and its metabolism

**DOI:** 10.3389/fonc.2022.982751

**Published:** 2022-08-25

**Authors:** Hui Jiang, Taku Kimura, Han Hai, Ryodai Yamamura, Masahiro Sonoshita

**Affiliations:** ^1^ Division of Biomedical Oncology, Institute for Genetic Medicine, Hokkaido University, Sapporo, Japan; ^2^ Department of Oral Diagnosis and Medicine, Graduate school of Dental Medicine, Hokkaido University, Sapporo, Japan; ^3^ Global Station for Biosurfaces and Drug Discovery, Hokkaido University, Sapporo, Japan

**Keywords:** cancer, *Drosophila*, genetics, metabolic reprogramming, drug discovery

## Abstract

Cancer is one of the most severe health problems worldwide accounting for the second leading cause of death. Studies have indicated that cancers utilize different metabolic systems as compared with normal cells to produce extra energy and substances required for their survival, which contributes to tumor formation and progression. Recently, the fruit fly *Drosophila* has been attracting significant attention as a whole-body model for elucidating the cancer mechanisms including metabolism. This tiny organism offers a valuable toolkit with various advantages such as high genetic conservation and similar drug response to mammals. In this review, we introduce flies modeling for cancer patient genotypes which have pinpointed novel therapeutic targets and drug candidates in the salivary gland, thyroid, colon, lung, and brain. Furthermore, we introduce fly models for metabolic diseases such as diabetes mellitus, obesity, and cachexia. Diabetes mellitus and obesity are widely acknowledged risk factors for cancer, while cachexia is a cancer-related metabolic condition. In addition, we specifically focus on two cancer metabolic alterations: the Warburg effect and redox metabolism. Indeed, flies proved useful to reveal the relationship between these metabolic changes and cancer. Such accumulating achievements indicate that *Drosophila* offers an efficient platform to clarify the mechanisms of cancer as a systemic disease.

## Introduction

Cancer ranks the second leading cause of death worldwide, and its disease burden continues to increase yearly (1). This malignant disease involves genetic alterations which induce various cancer hallmarks such as sustaining cell proliferation and invasion to promote cellular transformation (2). To date, preclinical studies have typically used cultured cells and mouse models to elucidate the cancer mechanisms and to identify numerous therapeutic agents. However, developing novel therapeutics still faces many challenges including low success rates in clinical trials, the high toxicity of therapeutic candidates and even approved drugs, and emerging resistance in patients (3). These challenges imply that it is inevitable to introduce additional approaches to complement the current efforts to clear the hurdles efficiently.

**Figure 1:**
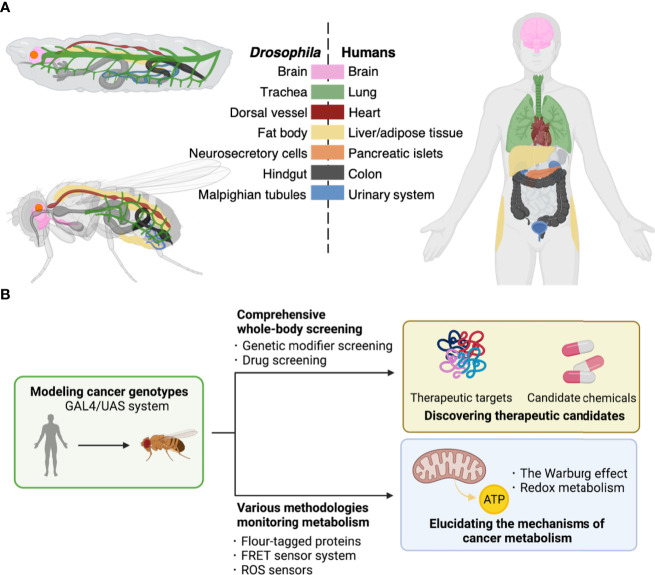
*Drosophila* platforms to study cancer and its metabolism. **(A)**, Corresponding tissues/organs regarding their structures and functions between *Drosophila* and humans. Each color indicates tissues/organs with similar functions among *Drosophila* larva (top upper left) and adult (bottom left), and human (right). Such similarities among metabolic pathways and physiological responses allow construction of fly models for human diseases of both cancers and metabolic disorders. **(B)**, The GAL4/UAS system enables induction of genes of interest in target fly tissues. These flies have allowed discovery of therapeutic targets including kinases and development of potent compounds for cancer treatment by comprehensive screenings (top). Furthermore, flies offer a useful toolkit including reporter lines to study cancer metabolism (bottom).

Here, we will introduce the fruit fly *Drosophila* as one of the ideal whole-body models to this end. *Drosophila* has a high rate of reproduction and low husbandry cost in laboratories. In addition, *Drosophila* is well-characterized for its genome with over 70% of disease-associated genes in humans (4, 5). Furthermore, flies show structural and physiological conservations in tissues/organs with mammals such as the brain, lung, heart, liver/adipose tissue, pancreatic islets, colon, and urinary system (**Figure 1A**). These similarities provide a powerful advantage in elucidating the mechanisms of tumorigenesis in specific organs. In light of modeling cancer genotype, flies offer a robust genetic toolkit to achieve precise genetic manipulation, which makes them a useful model organism in studies on cancer as a genetic disease. Indeed, there have been multiple fly models emerging, with single or multiple driver mutations to mimic cancer genotypes in patients (6). These models have allowed exploring the roles of such abnormalities in carcinogenesis and developing anti-cancer leads (**Figure 1B**) (6).

In addition to elucidating the mechanisms of cancer and to developing novel therapeutic strategies, flies also have contributed to delineating unique metabolic networks within cancer cells. Previous studies have demonstrated that tumor-associated metabolic reprogramming led by oncogenic mutations plays an important role in driving sustained cancer cell proliferation hence accelerating malignant progression (7). One of the first discoveries of such metabolic shift is aerobic glycolysis known as the Warburg effect, the vigorous glucose uptake to fuel glycolysis and secretion of lactate by cancer cells even in the presence of oxygen. Cancer cells intake extra glucose as compared with normal cells to produce extra energy and substances required for their survival (7, 8).

Meanwhile, the overproduction of reactive oxygen species (ROS) by dysfunctional mitochondria is another significant metabolic alteration in cancer cells attracting much attention these years. Excessive amounts of ROS cause cytotoxicity by inducing intracellular oxidative stress, which accumulates over time and ultimately leads to cell death (9). However, cancer cells have flexible responses to produce reducing equivalents against intracellular oxidative stress and foster cancer cell proliferation at last (8, 10). Hence, therapeutic strategies targeting metabolic vulnerabilities of cancer show the potential to become effective treatments and to combat drug resistance of cancer cells (11, 12). However, our limited knowledge has yet unraveled the metabolic programming in cancers, which prevents us from going further in identifying novel therapeutic candidates.

**Figure 2:**
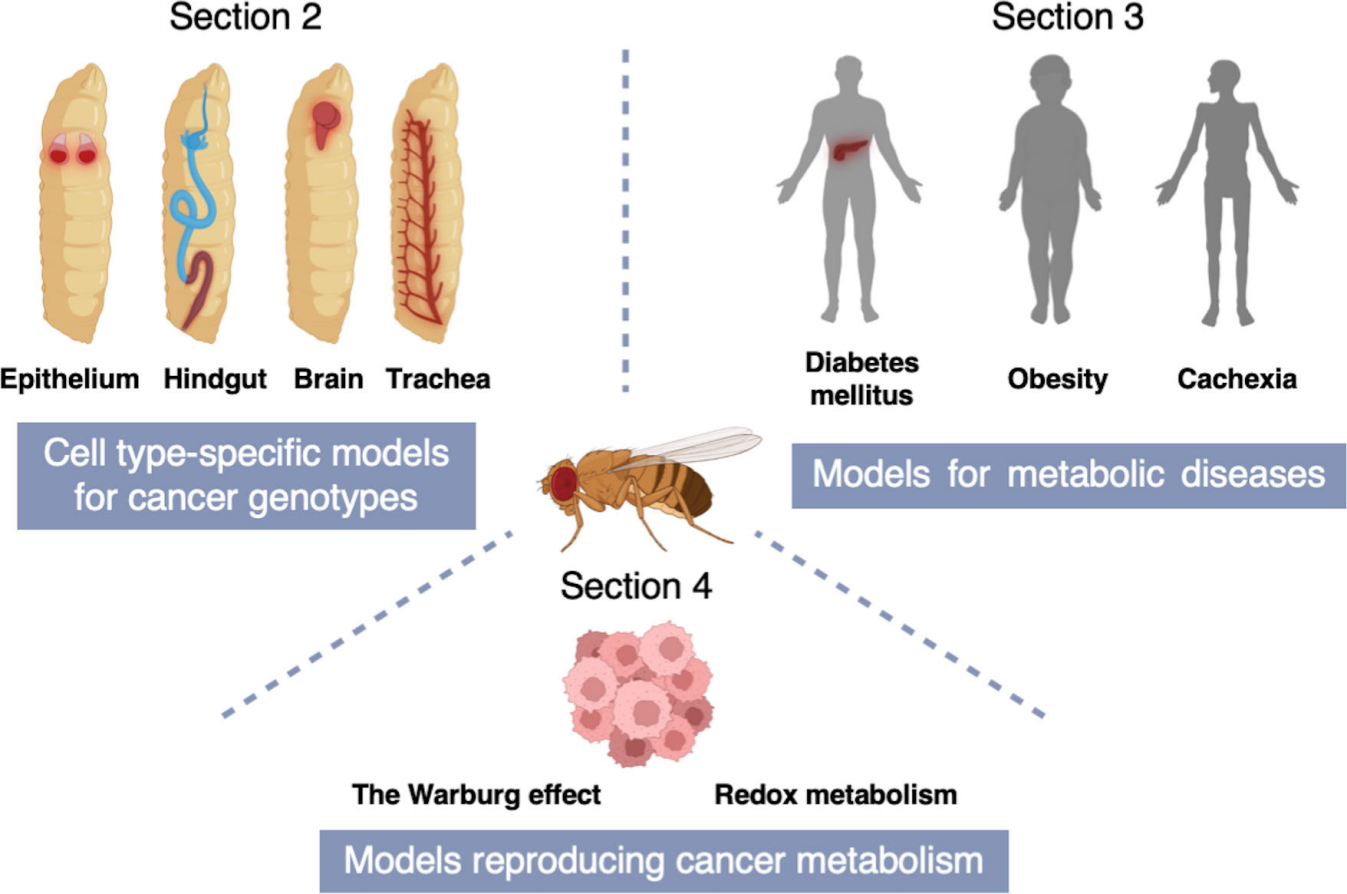
** **A schematic of the structure of this review. Section 2 describes cell type-specific models for cancer genotypes to mimic equivalent cancers in specific fly tissues [Adenoid cystic carcinoma (AdCC) and thyroid cancer (TC) in the fly wing disc epithelium; Colorectal cancer (CRC) in the fly hindgut; Non-small cell lung cancer (NSCLC) in the fly trachea; Glioblastoma (GBM) in the fly brain]. In Section 3, we introduce fly models for metabolic diseases including obesity, cachexia, and diabetes mellitus. Section 4 indicates fly models reproducing cancer metabolism which provide novel insights into the Warburg effect and redox metabolism.

In this review, we will first introduce fly models for various cancer genotypes (Section 2) and then introduce fly models for metabolic diseases including obesity, cachexia, and diabetes mellitus (Section 3). Lastly, we will put emphasis on fly studies that have provided novel insights into cancer metabolism (Section 4) (**Figure 2**).

## Fly models for various cancer genotypes

In this section, we introduce cell type-specific fly models for cancer genotypes (**Figure 2**).

### Adenoid cystic carcinoma

Adenoid cystic carcinoma (AdCC) is a rare gland tumor accounting for ~1% of all malignant tumors in the head and neck region and ~10% of all salivary gland cancers (13). AdCC generally grows slowly accompanied with perineural invasion, and more than 60% of the patients suffer local recurrence and/or distant metastasis (14). Such high recurrence and metastasis rates suggest that AdCC expands beyond surgical margins, causing hematogenous metastasis at early stages. The most common target organs of AdCC metastasis are lungs, bones, and livers (15).

Current treatment against AdCC includes radical surgical resection and adjuvant radiotherapy. Furthermore, clinicians often execute cytotoxic chemotherapy and targeted therapy against advanced AdCC. However, it is often difficult to confirm their therapeutic effect because AdCC responds to these treatments slowly. Even advanced AdCC sometimes becomes highly resistant to these treatments, which can lead to patients’ death (13). These circumstances lead to the long-term poor prognosis of patients. In fact, the 5-year rate of overall survival (OS) is around 70% but the long-term OS rate drops significantly (10- and 15-year OS: 54% and 37%, respectively).

As per mutational landscape, recent progress in genomic sequencing revealed that over 50% of AdCC have the *MYB*-*NFIB* fusion gene (16). This abnormality leads to overexpression of *MYB*, hence its transcriptional target genes associated with apoptosis (*API5*, *BCL2*, *BIRC3*, *HSPA8*, *SET*), cell cycle (*CCNB1*, *CDC2*, *MAD1L1*), cell growth and angiogenesis (*MYC*, *KIT*, *VEGFA*, *FGF2*, *CD53*), and cell adhesion (*CD34*) (17). These findings suggest that *MYB* activation promotes AdCC development, and products of these genes offer novel therapeutic targets for this cancer.

Recently, several research platforms such as human AdCC organoids and mouse models carrying patient derived xenograft (PDX) of AdCC have emerged. These achievements significantly contributed to revealing fundamental aspects of AdCC such as its pathogenesis and novel therapeutic targets (18–20). However, a high difficulty in obtaining sufficient AdCC samples due to its low availability has made it extremely difficult to accelerate further analyses on AdCC using these mammalian models.

To overcome this issue, Bangi et al. took a *Drosophila*-based research approach for a patient with advanced AdCC (21). They developed a patient-specific transgenic fly strain as a ‘personalized fly avatar’ that modeled the patient’s somatic mutations. To this end, they sequenced the patient’s genome and selected six major alterations including *MYB-NFIB* fusion, *NOTCH1* overexpression, and missense mutations of *FAT1*, *3*, *4* and *ERCC2*. Next, they introduced into the fly avatar their fly orthologs. Regarding *MYB-NFIB*, they utilized truncated human *MYB* which mimicked an N-terminal product lacking the C-terminal regulatory domain (**Table 1**).

**Table 1:** *Drosophila* models of various cancer genotypes and drug development using these strains.

Patient			*Drosophila*			Ref.
Cancer type		Genotype	Genotype	Phenotype	Therapeutic candidates	
Adenoid cystic carcinoma (AdCC)		*MYB*-*NFIB*, *NOTCH1* ^act^, *FAT1/3* ^missense^, *FAT4* ^missense^, *ERCC2* ^missense^	*ptc*>*MYB*-ΔC,*N*, *Kug* ^RNAi^,*ft* ^RNAi^, *Xpd* ^RNAi^	Cell proliferation, cell migration	A personalized combination therapy with **vorinostat** (a histone deacetylase inhibitor drug for cancer), **pindolol** (a non-selective beta blocker drug for high blood pressure), and **tofacitinib** (a JAK inhibitor drug for rheumatoid arthritis)	(21)
Thyroid cancer (TC)	Medullary TC (MTC)	*RET* ^M918T^	*GMR*-*dRet* ^M955T^	‘Rough eye’ with partially fused and inconsistent patterns of ommatidia	**ZD6474**, approved as **vandetanib** by FDA as the first targeted therapy for MTC	(22)
			*ptc*>*dRet* ^M955T^	Cell proliferation, cell migration	**AD80**, which was modified structure of AD57 to reduce its toxicity	(23)
				Cell proliferation, cell migration	**APS6-45**, which was modified structure of sorafenib to reduce its toxicity	(6)
	Papillary TC (PTC)	*CCDC6*-*RET*, *NCOA4*-*RET*	*ptc*>*CCDC6-RET*, *ptc*>*NCOA4*-*RET*	Cell proliferation, cell migration	A combination of **sorafenib** plus a WEE1 inhibitor **AZD1775**	(24)
Colorectal cancer (CRC)		*KRAS* ^G12V^, *TP53* ^inact^, *PTEN* ^inact^, *APC* ^inact^, *SMAD4* ^inact^	*byn*>*ras* ^G12V^,*p53* ^RNAi^, *pten* ^RNAi^,*apc* ^RNAi^, *Med* ^RNAi^	Cell proliferation, EMT, cell migration	A combination of the proteasome inhibitor **bortezomib** and the PI3K pathway inhibitor **BEZ235**	(25)
		*KRAS* ^G13A^, *TP53* ^inact^, *APC* ^inact^ *FBXW7* ^inact^, TGFBR2^inact^, *SMARCA4* ^inact^, *FAT4* ^inact^, *MAPK14* ^inact^, *CDH1* ^inact^	*byn*>*ras* ^G12V^,*p53* ^RNAi^,*apc* ^RNAi^, *ago* ^RNAi^,*put* ^RNAi^,*brm* ^RNAi^, *ft* ^RNAi^,*p38* ^RNAi^,*shg* ^RNAi^	Hindgut expansion	A combination of the MEK inhibitor **trametinib** and a bisphosphonate **zoledronate**	(26)
		*KRAS* ^act^, *APC* ^inact^	*esg*>*ras* ^G12V^,*apc* ^Q8/^ *apc*2^N175K^	Increased tumor burden	n/d	(27)
Non-small cell lung cancer (NSCLC)		*KRAS* ^G12V^, loss of *PTEN*	*btl*>*ras* ^G12V^,*pten* ^RNAi^	Cell proliferation, fly lethality	A combination of the MEK inhibitor **trametinib** and the HMG-CoA reductase inhibitor **fluvastatin**	(28)
		*EGFR* ^act^	*ppk*>*Egfr* ^A877T^	Tracheal epithelial cell malformation, larval death	A combination of the tyrosine kinases inhibitor **afatinib** and the JAK/STAT signaling inhibitor **bazedoxifene**	(29)
		*EGFR* ^act^, *KRAS* ^G12D^, *RAF* ^act^, *MAPK* ^act^, *PIK3CD* ^act^, *ALK* ^act^, *AKT* ^act^, *CTNNB*1^act^	*ppk*>*Egfr* ^A887T^,*ras* ^G12V^,*Raf* ^OE^, *Rolled* ^OE^,*Pi3K92E* ^OE^,*Alk* ^OE^, *Akt* ^OE^,*Arm* ^OE^	Tracheal epithelial cell proliferation and thickening, larval death	The MEK inhibitor **trametinib**	(30)
Glioblastoma multiforme (GBM)		*EGFR* ^act^, *PIK3CA* ^act^	*repo*>*dEGFR^λ^*,*dp110* ^CAAX^	Glial cell proliferation and invasion	A combination of the YAP/TAZ-TEAD transcriptional activation inhibitor **verteporfin** and the ACAT1 inhibitor **K-604**	(31–33)

*ptc*, *patched*; *GMR*, *glass multimer reporter*; *byn*, *brachyenteron*; *esg*, *escargot*; *btl*, *breathless*; *ppk*, *pickpocket*; *repo*, *reversed polarity*; EMT, epithelial-mesenchymal transition.

To test their effects on epithelial cells, the authors drove expression of these genes using the *patched* (*ptc*) gene enhancer/promoter in larval wing discs which proved useful to characterize cellular transformation (34). Upon induction of the transgenes, this fly avatar exhibited excessive proliferation of epithelial cells and decreased fly viability. They then conducted a large-scale screening of all FDA-approved drugs in this avatar by feeding them orally. Through this test they identified a three-drug cocktail as a therapeutic candidate to increase fly viability composed of vorinostat (the histone deacetylase inhibitor drug for cancer), pindolol (the non-selective beta blocker drug for high blood pressure), and tofacitinib (the JAK inhibitor drug for rheumatoid arthritis). Indeed, this cocktail led to disease stabilization and a partial metabolic response for 12 months in the patient. This study demonstrated that such a personalized approach using flies as a whole-animal platform can be useful in developing new treatments for AdCC.

### Thyroid cancer

Incidence of thyroid cancer (TC) has been increasing worldwide. TC represents the most common endocrine malignancy, accounting for 3.4% of all cancers diagnosed annually in the U.S (35). In recent years, TC can be detected in earlier stages than before because screening tests for TC have been becoming available in the clinical practice. However, mortality rate of TC has been increasing slightly and makes it a significant unmet clinical need (36).

TC mainly originates from endoderm-derived follicular cells or neural crest-derived C cells in the thyroid, and the majority of TC can be divided into two subtypes depending on their origins: papillary TC (PTC) and medullary TC (MTC). PTC originating from the former cells is the main subtype of TC accounting for 84% of all TCs, while MTC is relatively rare accounting for 4% of all TCs (37).

In terms of genetic background, > 30% of PTC and > 50% of MTC harbor activation of RET, a receptor tyrosine kinase (RTK) (38). Specifically, PTC carries *RET* fusion genes upon a chromosomal translocation to produce constitutively active RET proteins (37). Especially, two *RET* fusions *CCDC6*-*RET* and *NCOA4*-*RET* account for more than 90% of all PTC fusion genes (37, 39). Regarding MTC, *RET* is also the most frequently mutated gene followed by *RAS* mutations and *RET* or *ALK* fusions (37). Particularly Met918 to Thr (M918T), the amino acid substitution in the RET kinase domain, is one of the most common mutations in MTC. This alteration leads to conformational changes in RET protein decreasing its autoinhibition mechanisms causing phosphorylation even in the absence of its ligands such as brain-derived neurotrophic factor (BDNF) (38, 40).

Despite these molecular findings, developing novel therapeutics for PTC and MTC has been problematic due to the lack of efficient experimental tools. Additionally, RET inhibition turned out harmful causing severe adverse effects (41). Also, RET inhibitors suppress other tyrosine kinases which are structurally similar to RET resulting in unexpected systematic reactions (38, 41).

To overcome these problems, groups including us have developed and utilized fly models for TC genotypes, discovering novel therapeutics efficiently. For example, Vidal et al. introduced an active form of *Drosophila Ret* (*dRet* ^M955T^, analogous to the human *RET* ^M918T^) to adult fly eyes to generate *Glass multimer reporter* (*GMR*)-*dRet* ^M955T^ flies (**Table 1**) (22). This model displayed transformation of eye cells causing ‘rough eye’ phenotype due to cell proliferation. Using this model, they revealed that a tyrosine kinase inhibitor ZD6474 rescued this abnormality upon oral administration. Eventually, ZD6474 got approved in 2011 as the first targeted therapy vandetanib for MTC. This suggests that flies with cancer genotypes have potential to contribute to the development of therapeutics for human cancers.

Focusing on discovering compounds with higher anti-tumor effect than conventional kinase inhibitors, Dar et al. executed comprehensive chemical and genetic screenings in another fly model with MTC genotype (*ptc*>*dRet* ^M955T^; **Table 1**) (23). Kinase inhibitors such as RET inhibitors mentioned above typically interact with multiple targets beside their intended targets. This polypharmacological nature of a chemical affects various signaling pathways to modulate its efficacy and toxicity. Therefore, the authors attempted to optimize the polypharmacological profile of a kinase inhibitor. To this end, they developed an original chemical library targeting RET and other tyrosine kinases (91 in total) and found that one of the chemicals AD57 rescued tumorigenic phenotypes in *ptc*>*dRet* ^M955T^ flies efficiently. In this study, they also found in genetic screening that transformation in these flies was dependent largely on *Raf*, *Src*, and *S6K* (42). AD57 consistently inhibited these kinases, but it simultaneously inhibited *dTor* (a fly ortholog of human *mTOR*) which was an effector of Phosphoinositide 3-kinase (PI3K) and a suppressor of Raf (43). As a result of Raf deregulation hence dTor activation, AD57 caused high toxicity beside efficacy in flies. Accordingly, they modified the chemical structure of AD57 to generate AD80 which did not inhibit dTor. As expected, AD80 rescued fly lethality and cellular transformation including malformations of wing veins and cuticles more efficiently than AD57 without showing obvious toxicity. Of note, AD80 suppressed TC xenograft in mice dramatically (23). Recently, AD80 demonstrated a 100- to 1000- fold higher anti-tumor effect on TC cell lines than other multikinase inhibitors approved for RET-dependent cancers including sunitinib, sorafenib, vandetanib, and cabozantinib (44). These studies prove that flies provide potential preferred and non-preferred targets of kinase inhibitors. Applying these achievements will reveal mechanisms of adverse effects of various anti-cancer drugs and eventually lead to establishment of potent anti-cancer drugs with reduced toxicity.

In order to develop therapeutic compounds that preserve their anti-tumor effect while reducing their toxicity in a more rational manner, we established a novel method for developing therapeutics by utilizing comprehensive chemical and genetic modifier screens in *ptc*>*dRet* ^M955T^ flies (**Table 1**) (6). We first screened in this model all kinase inhibitor drugs approved by FDA for cancer therapy at the time of 2016, and confirmed that sorafenib showed the strongest but only marginal effects. In clinical practice sorafenib has given benefits to MTC patients, but severe adverse effects emerge such as skin damage, diarrhea, alopecia and even fatality in patients (45, 46).

We thus attempted to determine the cause of this toxicity through comprehensive chemical and genetic modifier screens of the kinome network in *ptc*>*dRet* ^M955T^ flies. First, we developed a library of sorafenib analogs by their chemical synthesis and fed them orally to *ptc*>*dRet* ^M955T^ flies, finding out several derivatives with improved efficacy measured by fly viability as a readout. Then, we executed genetic modifier screening (199 in total, covering more than 80% of all fly kinases) in the presence of sorafenib or such derivatives to elucidate the mechanisms of their efficacy and toxicity. Interestingly, inhibiting one of the sorafenib targets Lk6 [a fly ortholog of human Mitogen-activated protein (MAP) kinase-interacting serine/threonine-protein kinase (MKNK)] by removing one copy of *Lk6* gene in these flies (*ptc > dRet* ^M955T^,*Lk6 ptc > dRET* ^M955T^, *Lk6* ^−/+^) caused complete lethality in the presence of sorafenib. Control *Lk6* ^−/+^ flies presented almost 100% survival, therefore these results indicate that sorafenib has LK6 as an ‘anti-target’ whose inhibition accounts for its toxicity. These findings led us to derivatize sorafenib further to generate APS6-45 which *in silico* modeling predicted to have significantly reduced binding capacity with MKNK but not RET as compared with sorafenib. As expected, APS6-45 suppressed growth of human MTC cell line TT and its xenograft in mice without detectable toxicity, suggesting that APS6-45 offers a novel therapeutics for treating MTC. As such, this ‘rational polypharmacology’ integrating multiple fly screening platforms with computational chemistry with medicinal chemistry can accelerate development of novel high-efficacy and low-toxicity drugs.

As per PTC, a previous study generated fly models for PTC genotypes including *CCDC6*-*RET* and *NCOA4*-*RET* fusions, both of which are frequently observed in the patients (**Table 1**) (24). The authors employed these fly models to identify compounds for PTC treatment and to validate functional differences between two types of human *RET* fusions. These flies exhibited tumorigenic phenotypes in wing discs including cell migration and delamination. Of importance, flies with human *NCOA4*-*RET* fusion showed more severe phenotypes than those with human *CCDC6*-*RET*, consistent with outcome of PTC patients carrying distinct genetic abnormalities. In this paper, the authors revealed key roles of MAP kinase (MAPK) signaling pathway in PTC development. Notably, they also identified that these *RET* fusions activated distinct signaling pathways; *NCOA4*-*RET* but not *CCDC6*-*RET* activated Hippo and PI3K pathways. Furthermore, chemical screenings in these flies for FDA drugs and experimental small molecules (55 in total) revealed that these fusions conferred different drug sensitivity. Specifically, sorafenib and cabozantinib rescued lethality of *NCOA4*-*RET* flies, whereas gefitinib and vandetanib rescued that of *CCDC6*-*RET* flies. Therefore, they concluded that these two *RET* fusions activated different signaling pathways to promote transformation and determine distinct sensitivity to clinically relevant drugs. Their achievements indicate that fly platforms are useful not only for identifying therapeutic targets and chemicals against cancers but also for analyzing functions of human genes.

Collectively, transgenic flies successfully unveiled the fundamental effects of abnormalities in TC genome on cellular characteristics. Generating and testing more TC models will accelerate comprehensive determination of TC pathogenesis and novel therapeutics.

### Colorectal cancer

Colorectal cancer (CRC) is the third most diagnosed cancer and the second leading cause of cancer death globally accounting for 10% of total cancer cases and 935,000 deaths in 2020, respectively (47). Our previous work based on mammalian models has given us important insights into the CRC mechanisms. For example, we demonstrated that PGE2-EP2 and NOTCH-ABL-TRIO-RHO pathways promote CRC initiation and progression, respectively (48–53). Also, we identified the invasion/metastasis-suppressing *Aes* gene which inhibits NOTCH signaling (54–57).

Although plenty of previous studies including them have deepened our understanding of cancer signaling pathways, tackling CRC remains to be an important challenge. To solve this, *Drosophila* has proven a powerful whole-body model due to its significant similarities in both physiology and morphology of the digestive tract to mammalians (**Figure 1A**) (58). The fly gut has similar functions to its mammalian counterparts to digest food, absorb nutrients, and execute the first-line defense against infection by innate immunity (59, 60). Based on their functions, the fly gut is divided into three parts: the foregut, midgut, and hindgut (61). Among them, the midgut is regarded as useful to study the contribution of signaling pathways and metabolism in CRC, because its architecture resembles digestive tracts of mammals (58, 62).

On the other hand, CRC onset has several related signaling pathways: *WNT*, *SMAD4*, *KRAS*, *PIK3CA*, and *TP53* (63). Inactivation of the *Adenomatous polyposis coli* (*APC*) gene, a tumor suppressor in the WNT signaling pathway, is the most common mutation in CRC occurring in 80-90% of patients (64). As the second most common mutation, approximately 50% of CRCs are homozygous for loss-of-function mutations in the *TP53* tumor suppressor gene, followed by gain-of-function mutations in the *KRAS* oncogene in around 40% of CRCs (65, 66). Their identifications led to genetic manipulation of two or more of them in combination *via* mouse genetics (67). Unfortunately, genetically engineered mouse models (GEMMs) with complex genotypes require enormous resources to generate and maintain. In this regard, *Drosophila* offers advantages because modeling multiple mutations is easy in flies. Hence, as we will state in this section, fly models for CRC genotypes contributed to unraveling the complexity of CRC regarding disease metabolism and drug response complementarily with mammalian models.

Modeling recurrent mutations in CRC, a group induced five cDNAs and knockdown siRNAs as transgenes in the fly hindgut, including *KRAS* (fly *ras*), *TP53* (*p53*), *PTEN* (*pten*), *SMAD4* (*Med*), and *APC* (*apc*) by using the *byn* (*brachyenteron*) enhancer/promoter (**Table 1**). These genetic modifications resulted in cellular transformation recapitulating hallmarks of human CRC including cell proliferation, disruption of the epithelial architecture, EMT, migration and dissemination to distant sites. By using these multigenic flies, the authors identified the mechanism of resistance against a PI3K/mTOR inhibitor BEZ235. They further discovered two-step therapy using bortezomib (the proteasome inhibitor) and BEZ235 to overcome this resistance in this model. This treatment was also effective in a CRC cell line DLD1 carrying a similar mutational signature to the multigenic flies, as well as in its xenografts in mice. This study provided important insights into the use of flies as a handy platform for rapid and large-scale functional exploration of human cancer genomes as well as drug discovery (25). Moreover, the authors published another milestone paper where they developed a personalized fly model of a patient with refractory metastatic CRC harboring *KRAS* mutation (**Table 1**) (26). In FDA drug screening, combination between trametinib [the drug targeting Mitogen-activated protein kinase kinase (MEK)] and zoledronate (a bisphosphonate) significantly suppressed anterior expansion of the hindgut. This treatment gave a significant response to the patient reducing the tumor volume by 45%. Notably, CRC remained stable for 11 months in the patient (26).

Following these studies, another group developed novel high-throughput assays for quantifying tumor burden (27). They reported two methods to evaluate proliferation of transformed cells. One method is to use a simple software they developed in ImageJ Fiji to automatically analyze the area that transformed cells occupy. Another is to use luciferase as a reporter to determine the number of transformed cells. By these two methods, they reported increased tumor burden in a fly model for CRC genotype carrying the *esg* (*Escargot*)-GAL4 driver to induce *ras* ^G12V^ and *apc* ^LOF (Loss of function)^ transgenes specifically in intestinal stem cells (ISCs) (**Table 1**). Besides, another study designed an *in silico Drosophila* model for CRC genotype based on data of cell type-specific RNA-seq on FlyGut-seq database (68). They constructed a computational framework for the fly midgut, which successfully elucidated cell fate, validated drug cytotoxicity, and devised a personalized treatment candidate. To summarize, *Drosophila* is a useful preclinical whole-animal model due to its multiple applications in CRC studies.

Different from *byn* cells throughout the hindgut and a subset of posteriorly derived visceral muscles (26), another driver *esg*-GAL4 is frequently used in fly CRC studies. *esg*-GAL4 is active in progenitor cells in the posterior midgut, which are also known as ISCs (27). It has not been declared in papers that inducing transformation in fly ISCs mimics CRC tumorigenesis derived from mutated stem cells in patients. However, modeling CRC mutations in fly ISCs provided important clues of metabolic reprogramming in stem cells with tumorigenic potential (62, 69–71). Specifically, a study revealed that activation of *yorkie* (*yki*), a fly ortholog of the human oncogene *YAP1*, leads to proliferation of ISCs *via* upregulation of insulin/insulin-like growth factors (IGF) signaling and glycolysis (69). In addition, studies have shown that elevated lactate concentration caused by Ras/Raf activation in ISCs caused Warburg effect-like metabolic changes in transformed cells to induce proliferation of transformed cells (62, 70). Another study showed that tumor-like ISCs induced by Notch depletion proliferated and generated ROS, while ISCs with reduction of both Notch and β-integrin caused metastasis and ROS (71). Overall, these studies demonstrated that flies are practical in mechanistic analyses and drug discovery of CRC.

### Lung cancer

Lung cancer is the top cause of global cancer mortality with a rising incidence (72). With a large number of diagnoses each year, reported 5-year survival remains ~15% for all patients and less than 4% for those with distant metastasis. Unfortunately, only minimal improvement has been made in these dismal statistics over the past decades (73). Because of such disappointing prognosis and significant systemic toxicities of even approved treatment, developing novel therapies has remained one of the major goals in lung cancer research.

To solve this issue, *Drosophila* has been used to model lung cancer genotypes to develop novel therapeutics recently (29), breaking the long-standing underestimation of the potential of *Drosophila* in lung cancer research (74). In fact, *Drosophila* is devoid of lungs. However, the respiratory systems in flies and mammals share lots of structural and physiologic similarities (**Figure 1A**) (75). For example, fly models of chronic lung diseases such as asthma and chronic obstructive pulmonary disease (COPD) are available in the field, demonstrating the feasibility of modeling lung diseases in flies by mimicking their genotypes in the respiratory system (76).

Non-small cell lung cancer (NSCLC) accounts for approximately 84% of all primary lung cancers (77). *KRAS* and *Epidermal growth factor receptor* (*EGFR*) are the two most common identifiable drivers, whose mutations cover 50%–60% of NSCLC cases (78, 79). Based on this information, several groups developed fly models of NSCLC genotypes and fly-based platforms for processing high-throughput chemical screening (28–30). For example, Levine and Cagan developed the first *Drosophila* lung cancer model by targeting *ras* ^G12V^ alone or in combination with *PTEN* knockdown to the fly tracheal system using the *breathless* (*btl*)-GAL4 driver (**Table 1**). In this model, *ras* ^G12V^ induced tracheal proliferation and fly lethality in the larval or pupal stage. Using this lethality as a readout, the authors screened the library of FDA-approved drugs and identified inhibitors of MEK and HMG-CoA (3-hydroxy-3-methylglutaryl coenzyme A) reductase as potential therapeutics (28).

While, other teams used the *pickpocket* (*ppk4*)-GAL4 driver to induce constitutive activation of *EGFR* in the fly trachea, causing malformation of tracheal epithelium and larval death. Among ~1,000 FDA-approved drugs, only tyrosine kinase inhibitors (TKI) afatinib, gefitinib, and ibrutinib rescued *EGFR*-induced larval lethality. By utilizing the fly-based whole-animal screening, they identified synergistic anti-tumor effects of a combination of afatinib and bazedoxifene, a novel GP130/STAT3 pathway inhibitor. These findings suggested therapeutic benefits by simultaneously blocking EGFR and JAK/STAT signaling in NSCLC (**Table 1**) (29).

Even after establishment of such fly-based high-throughput screening systems, it was still unclear if other driver mutations also caused transformation. To answer this question, the authors further developed modular *Drosophila* models for a larger number of human lung cancer oncogenes including *Egfr*, *ras* ^G12V^, *Raf*, *Rolled* (a fly ortholog of human *MAPK*), *Pi3K92E* (*PIK3CD*), *Alk*, *Akt*, and *Arm* (*CTNNB1*) (**Table 1**) (30). On the other hand, they established two complementary readouts which were simple, reliable, and adaptive to the needs of high-throughput screening. One of the readouts was rescue of fly lethality, and the other was reduction of a quantifiable tumor mass (30). This workflow demonstrated a possibility of *Drosophila* to provide various high-throughput screening measures and thus novel lung cancer treatments.

### Glioblastoma

Gliomas, especially glioblastoma multiforme (GBM), is the most common primary malignant brain tumor in adults (80). The incidence of GBM is estimated at 3.2 per 100,000 population in the United States per year. The 5-year relative survival rate for this cancer is only 5%, making it one of the most deadly and recalcitrant tumors of all malignant solid tumors (81). In addition, even with surgical resection as the standard treatment, patients with GBM have a poor prognosis with a median survival of only 15 months (82). Considering such poor prognosis, developing effective therapies for GBM treatment is an urgent clinical need for improving clinical outcome.

According to genomic profiling including The Cancer Genome Atlas project, the most common genetic alteration for GBM is overexpression of *EGFR* (altered in 30-40% of total cases) and *PIK3CA* (8-10%) (83). Undoubtedly, identification of these key effectors involved in GBM are critical clues to develop novel measures for diagnosis and treatment (**Table 1**).

There are several studies demonstrating that the mechanisms of neural development are remarkably similar between flies and humans (**Figure 1B**) (84). Combined with the advantages of fly genetics, such similarities make flies an effective tool to model genotypes of gliomas to delineate their pathogenesis (85).

To investigate genetic basis and to determine novel therapeutic targets of GBM, Read et al. developed a fly model *repo*>*dEGFR* ^λ^,*dp110* ^CAAX^ based on *reversed polarity* (*repo*)*-*GAL4-driven co-overexpression of active forms of *Drosophila EGFR* (*dEGFR* ^λ^) and *PIK3CA* (*dp110* ^CAAX^) in glia. These alterations induced neoplasia causing proliferation and invasion of glial cells seen in human GBM (31). Thus, this fly model has been widely used in GBM studies and has brought novel insights into the molecular mechanisms of GBM (**Table 1**) (32, 86, 87).

Another group found that overgrowth and invasion of glial cells happen upon overexpression of other RTKs including *Pvr* [a fly ortholog of platelet-derived growth factor receptor (PDGFR)/vascular endothelial growth factor receptor (VEGFR)], *htl* [a fibroblast growth factor receptor (FGFR) ortholog], and *InR* (an insulin receptor ortholog) (86). Besides, they demonstrated that administration of the EGFR inhibitor drug gefitinib, the PI3K inhibitor wortmannin, and the Akt inhibitor triciribine can revert EGFR/PI3K-induced transformation (86).

After these studies, a kinome-wide genetic screening was conducted in *repo*>*dEGFR* ^λ^,*dp110* ^CAAX^ flies to discover effectors required for RTK- and PI3K-dependent neoplastic transformation. This test clarified that overexpression of right open reading frame (RIO) kinase driven by mTor-complex-2 (TORC2)-Akt signaling promoted cell proliferation and survival, which gives novel therapeutic opportunities for GBM (87). In the same model, *yki* was overexpressed in neoplastic glia, and its knockdown suppressed glial proliferation (32). This finding raises an FDA-approved liposomal formulation of verteporfin as a novel therapeutic option for EGFR-driven GBMs as it suppresses transcriptional activity of YAP/TAZ which are mammalian orthologs of fly *yki* (**Table 1**) (32). However, it is possible that abnormalities in multiple RTKs limit efficacy of therapies targeting a single RTK. Therefore, combining several RTK inhibitors can offer more effective treatment for GBM than monotherapy (88).

Accumulating evidence indicates that metabolic reprogramming in the brain is a critical factor in the transition from non-neoplasm to neoplasm including GBM. Therefore, targeting essential metabolic pathways in glia may provide new therapeutic opportunities for GBM treatment (89). In *repo*>*dEGFR* ^λ^,*dp110* ^CAAX^ flies, glia-specific knockdown of four genes essential for glial metabolism [*ALDOA* (*Aldolase* in flies), *ACAT1* (*CG8112*), *ELOVL6* (*Baldspot*), and *LOX* (*Lox*)] partly rescued glioma-induced phenotypes such as shorter lifespan and bigger tumor size. Of these four the authors especially focused on *ACAT1* which plays a role in regulating endoplasmic reticulum-cholesterol homeostasis and lipid metabolism. Then they found that silencing *ACAT1* maintained cholesterol homeostasis, and prevented brain hypertrophy and glioma trait-induced shortening of fly lifespan (**Table 1**) (33).

While, impaired insulin function leads to abnormal glucose metabolism and mitochondrial dysfunction (90). Based on the same fly model mentioned above, glioma-secreted *Imaginal morphogenesis protein-late 2* (*ImpL2*) was found to inhibit insulin pathway activity, which caused synaptic loss and consequently promoted neurodegeneration. Restoring insulin signaling in neurons by overexpressing *Rheb* (activation of insulin/TOR/S6K signaling pathway) partially rescued neurodegeneration and mortality of the model (91).

More than using flies to identify novel therapeutic targets, the utilization of the above-mentioned fly model verified that mitochondrial PTEN-induced kinase 1 (PINK1), a regulator of the Warburg effect, turned out to suppress GBM growth (92). *PINK1* overexpression attenuated GBM traits in both flies and orthotopic xenografts of human U87 cells in mice. In summary, these studies on flies have unraveled part of the mechanism by which significant alterations in metabolic pathways in cancer cells are associated with the onset and progression of GBM.

## Fly models for metabolic diseases

Similarities among metabolic pathways and physiological responses between *Drosophila* and humans makes flies a useful whole-animal model for not only genetic diseases but also metabolic disorders (93, 94). In this section, we describe fly models to mimic metabolic disorders. In particular, we will focus on diabetes mellitus (DM), obesity, and cachexia introducing contributions of their fly models to understand these diseases (**Figure 2**).

### Diabetes mellitus

Regarding sugar metabolism, different from mammals, flies take up simple sugars passively from the digestive tract into the fat body where they are converted to trehalose. Hence, trehalose is the primary circulating sugar in insects instead of glucose (95, 96). Despite this difference in substances, flies share functionally similar mechanisms with mammals to regulate sugar homeostasis through conserved pathways: *Drosophila* secretes an insulin equivalent insulin-like peptides (Ilps) and a glucagon analog adipokinetic hormone (AKH) to respond to high and low levels of circulating sugar, respectively (97–100). These facts generated an idea of modeling DM in flies.

Type 1 DM (T1DM) arises from the destruction of insulin-producing β-cells of the pancreas which results in decreased or complete loss of insulin. In flies, insulin-producing cells (IPCs) in the brain are equivalent to the β-pancreatic islet cells in mammals (**Figure 1B**). Upon ablation of IPCs, flies reproduced T1DM-like phenotypes such as growth defects and developmental delay (98, 99).

On the other hand, Type 2 DM (T2DM) is a disease of insulin resistance; namely hyperglycemia persists despite the presence of high levels of circulating insulin. Interestingly, feeding a high-sugar diet (HSD) caused insulin resistance in flies generating diabetic-like states such as hyperglycemia even with high levels of Ilps (101).

Epidemiological studies have shown an increased risk of several types of cancer in DM patients including pancreas, liver, breast, colorectal, urinary tract, and female reproductive organs (102). Hence, there have been studies to reveal the relationship between DM and cancer, and flies contributed to the discovery that HSD boosted tumor progression. One of the examples is that feeding flies with HSD promoted EGFR-driven epithelial neoplasia and metastasis through lactate dehydrogenase (LDH)-dependent aerobic glycolysis (103). Therefore, flies are practical toolkits to simultaneously reproduce tumorigenesis and systemic metabolic disorders to explore their mutual mechanisms.

### Obesity

Regarding the cause of T2DM, obesity induced by caloric excess is a triggering factor for insulin resistance-associated diabetes. In fact, 55% of T2DM patients are obese (104). Consistently, a fly model for HSD-induced T2DM manifested also obesity as determined by accumulating fat within the body (101). In these flies, HSD also triggered alterations in insulin signaling, lipogenesis, and gluconeogenesis. Therefore, this model not only revealed pathological relationships between multiple metabolic disorders but also gave rise to novel therapeutic candidates such as Gomisin N, which relieves the endoplasmic reticulum stress to be a potential agent for preventing and treating obesity (105).

So far, several epidemiological studies provided strong evidence of an association between obesity and increased risk of various cancers (106). To understand the interplay between obesity and cancer, a study induced obesity by HSD in flies modeling activation of multiple oncogenes such as *Ras* and *Src* (107). In this study, the active form of fly *ras* and a null allele for fly *C-terminal Src kinase* (*Csk*) induced tumors. Intriguingly, HSD caused the *ras1* ^G12V^;*csk* ^−/−^ tumors to grow more aggressively than normal diet. Simultaneously, the authors demonstrated that Ras/Src-activated cells efficiently responded to nutritional signals of a SIK-Yki-Wg-InR signaling circuit and ensured tumor growth upon nutrient-rich conditions including obesity (107, 108). As such, studies with flies have significant potential in elucidating the mechanisms by which obesity influences development and progression of cancer.

### Cachexia

Another cancer-related metabolic dysfunction cachexia is a multifactorial wasting syndrome that contributes to the clinical deterioration in patients with advanced cancer. Cachexia is characterized by weight loss, skeletal muscle wasting, and atrophy of the adipose tissue (109). Recently, some reports included *Drosophila* to recapitulate cachexia-like systemic wasting to obtain insights into the cachexia mechanisms (69, 110).

For example, a study developed *scrib* ^−/−^, *ras* ^G12V^ tumors in flies. The authors found robust wasting of adipose and muscle tissues in flies developing tumors, which resembled cancer cachexia in patients (110). Another study established a model for systemic organ wasting in adult flies by overexpressing *yki* using *esg*-GAL4 driver active in ISCs (69). Both studies stated that insulin signaling was impaired in transformed cells demonstrating the central role of tumor-induced insulin resistance in cachexia.

### Metabolic diseases and cancers

In addition to these models, flies turned out to be useful in modeling hepatic metabolic diseases, neurodegenerative diseases, and other types of metabolic dysfunctions (94, 111). These achievements come from high conservation of metabolic pathways between *Drosophila* and mammals. Therefore, it is possible to reproduce characteristic alterations in cancer metabolism in fly models of cancer genotypes to elucidate their mechanisms and their impact on each disease.

As one of such reactions in cancer, angiogenesis under hypoxic stress is an adaptive strategy in tumor progression to meet its metabolic needs (112). In analogy to human blood vessels, the fly tracheal system plays similar roles in transporting oxygen to internal organs (113). Previous studies showed that *Drosophila* transformed cells suffered from oxygen shortage similar to human cancers. Interestingly these cells released pro-tracheogenic factors, which led to identification of tracheogenesis as a novel tumor hallmark in flies (112). Therefore, *Drosophila* offers also a convenient whole-body organism to determine metabolic reprogramming in cancers.

*Drosophila* models for metabolic disorders are not only valuable for elucidating pathogenesis of these metabolic disorders but also able to contribute to cancer research. Several metabolic disorders such as obesity and DM have an important mutual influence on specific types of cancers (102, 114, 115). Intriguingly, up to 80% of pancreatic cancer patients are either hypoglycemic or diabetic in a presymptomatic phase. Therefore, new-onset diabetes is a potential clue to early diagnosis of pancreatic cancer (116). Indeed, metabolic disorders and cancer are too complex to recapitulate in mammalian models simultaneously. However, flies have provided a possibility to combine two models in one organism and have promoted understanding of the fundamental associations between metabolic diseases and cancers as discussed above. Moreover, flies contributed to elucidating the cachexia mechanisms such as cancer-host interactions. To summarize, fly studies provide us with simple and effective ways to explore critical insights not only of cancer development and progression but also of the connections between metabolic diseases and cancers.

## Cancer metabolism revealed by fly studies

In this section, we present fly models for studying cancer metabolism (**Figure 2**).

### The Warburg effect

Glucose metabolism is essential for cells to produce adenosine triphosphate (ATP) as an energy source to maintain their homeostasis and activity. In the process of glucose metabolism, normal cells break glucose into pyruvate in the cytosol by glycolysis, putting pyruvate into the tricarboxylic acid (TCA) cycle, also termed as Krebs cycle in mitochondria where pyruvate further gets metabolized (oxidized) into carbon dioxide and ATPs. It is well known that this glucose metabolism pathway is changeable depending on oxygen. In the presence of sufficient oxygen, most types of cells produce ATP through the TCA cycle and further steps of glucose metabolism including oxidative phosphorylation in mitochondria. Through this process, cells can generate 36 ATP molecules per one glucose molecule. Normally, cells obtain oxygen constantly from the blood circulation. On the other hand, cells under hypoxic conditions where oxygen is scarce largely count on glycolysis yielding lactate and just two ATPs per one glucose molecule. Therefore, in light of ATP production, glycolysis is far from an efficient strategy compared with oxidation of pyruvate through the following process in mitochondria. Intriguingly, Warburg discovered that cancer cells tended to employ glycolysis to produce energy even in the presence of sufficient oxygen, publishing his findings as the ‘Warburg effect’ (117).

In the process of the Warburg effect, LDH plays an essential role in promoting glycolysis. Human LDH enzymes are encoded by four distinct genes (*LDHA*, *LDHB*, *LDHC*, and *LDHD*). Among them, LDHA primarily converts pyruvate to lactate. Meanwhile, *Drosophila* has one *LDH* gene *ImpL3*, whose product functions similarly to mammalian LDH. Therefore, it seems reasonable to study ImpL3 in flies to effectively understand essential functions of human LDH in tumorigenesis.

In human cells where glucose metabolism is active, glyceraldehyde-3-phosphate dehydrogenase (GAPDH) generates nicotinamide adenine dinucleotide hydrogen (NADH) from nicotinamide adenine dinucleotide (NAD_+_) in glycolysis. NADH is an essential molecule for the forthcoming process, oxidative phosphorylation, to generate additional ATPs. In this process, reducing the amount of NAD^+^ results in decelerating the glycolytic process leading to growth restriction (118). In order to compensate for the shortage of NAD^+^, cancer cells largely use NAD+ production by LDH which oxidizes NADH and produces NAD^+^ in the process of converting pyruvate into lactate (119). Therefore, human LDH is responsible for maintaining the NAD^+^/NADH redox balance in highly glycolytic cells such as cancer cells.

Intriguingly, flies have similar compensation mechanisms in glucose metabolism as in human cells. For instance, larvae lacking *ImpL3* are still able to produce lactate by accelerating glycerol-3-phosphate (G3P) production with increased activity of G3P dehydrogenase 1 (GPDH1), which allows normal larval development. Therefore, GPDH1 regulates the NAD^+^/NADH redox balance and ATP level in larvae (120). Given the similar glycolytic processes and the conserved functions between ImpL3 and LDH, we speculate that flies give us clues to understand the fundamental mechanisms of the Warburg effect.

In recent years, several fly models for cancer genotypes have exhibited unique cell metabolism in transformed cells that are seen also in human cancer cells. Next, we introduce examples which give insights into the relationship between glucose metabolism and cancer (**Table 2**).

**Table 2:** *Drosophila* models reproducing cancer metabolism.

Human Gene	*Drosophila* Genotype	GAL4 Driver		Metabolic Phenotype		Mode of action	Assessment methodology		Ref.
*NOTCH* ^act^	*Nicd*	*ptc* (wing disc)		Upregulation of glycolysis associated genes including *ImpL3*		Upregulation of N transcriptional activity	ChIP assay with α-Su(H) antibody, mRNA measurement by qPCR		(121)
*PDGF/VEGF receptor* ^act^	*Pvr* ^act^	*dpp* (wing disc)		Upregulation of ImpL3		Stabilization of Hifα	*LDH*-*GFP* reporter		(122)
*HIPK* ^act^	*Hipk* ^OE^	*dpp* (wing disc)		Upregulation of glycolysis associated genes including *ImpL3*		Upregulation of dMyc	mRNA measurement by qPCR, *LDH*-*GFP* reporter, FRET glucose sensor		(123)
*COX7A* ^inact^	*COX7a* ^RNAi^	*ey* (eye disc)		Upregulation of ImpL3, Increased glucose uptake and level of intracellular lactate		Inhibition of mitochondrial ETC	*LDH*-*GFP* reporter, FRET glucose/lactate sensor		(124)
*DLG1* ^inact^	*dlg* depletion		*en* (wing disc)	Elevated ROS		Loss of cell polarity	DHE, DCFH-DA		(125)
*FLT1* ^act^	*Pvr* ^act^		*dpp* (wing disc)	Elevated ROS		Glycolytic tumor	DHE, *GstD*-*GFP* reporter		(126)
*KRAS* ^G12V^,*SCRIB* ^inact^	*ras* ^G12V^,*scrib^−/−^*		*en* (eye disc)	Elevated ROS		Loss of cell polarity	DHE, DCFH-DA		(127)
n/d	*brat* ^RNAi^		*da* (brain stem cells)	Elevated ROS		Brain stem cell tumor	ROS sensor CellRox		(128)
*KRAS* ^G12V^,*SCRIB* ^inact^	*ras* ^G12V^,*scrib^−/−^*		*ey* (eye disc)	Elevated ROS/ Reduced ROS under *ras* ^G12V^ regulation		Loss of cell polarity	MitoSOX		(129)
*MYC* ^act^,*PI3K* ^act^	*dMyc* ^OE^,*Pi3K92E* ^OE^		*hh* (wing disc)	Elevated ROS		Field cancerisation	DHE		(130)
n/d	*brat* ^RNAi^		*pnt*, *ase* (brain stem cells)	Elevated ROS		Brain stem cell tumor	ROS sensor CellRox		(131)
*HIPK* ^act^	*Hipk* ^OE^		*dpp* (wing disc)	Elevated ROS		Accumulated hyperpolarized mitochondria	DHE		(132)
*KRAS* ^G12V^	*ras* ^G12V^		*esg* (intestinal stem cells)	Elevated ROS/ Reduced ROS under *ras* ^G12V^ regulation		Intestinal stem cell tumor	ROS sensor RoGFP2		(70)
*BRAF* ^act^	*Raf* ^act^		*esg* (intestinal stem cells)	Elevated ROS	Intestinal stem cell tumor			DHE	(133)
*YAP1* ^act^	*yki* ^OE^		*GMR*,*ey*,*dpp* (adult eye)	Elevated ROS	Cardiac dysfunction			DHE	(134)
*NOTCH* ^inact^,*ITGB1* ^inact^	*N* ^RNAi^,*mys* ^RNAi^		*esg* (intestinal stem cells)	Elevated ROS	Intestinal stem cell tumor			DHE, MitoSOX, *GstD*-*GFP*	(21)
*NOTCH* ^act^	*N* ^OE^		*1407* (brain)	Elevated ROS	Brain tumor			DCFH-DA	(135)

ETC, Electron transport chain; Su(H), Suppressor of Hairless ISC, intestinal stem cell; BSC, brain stem cell; act, activation; inact, inactivation; OE, overexpression; n/d, not determined. *: Authors did not mention its ortholog in humans.

**Figure 3:**
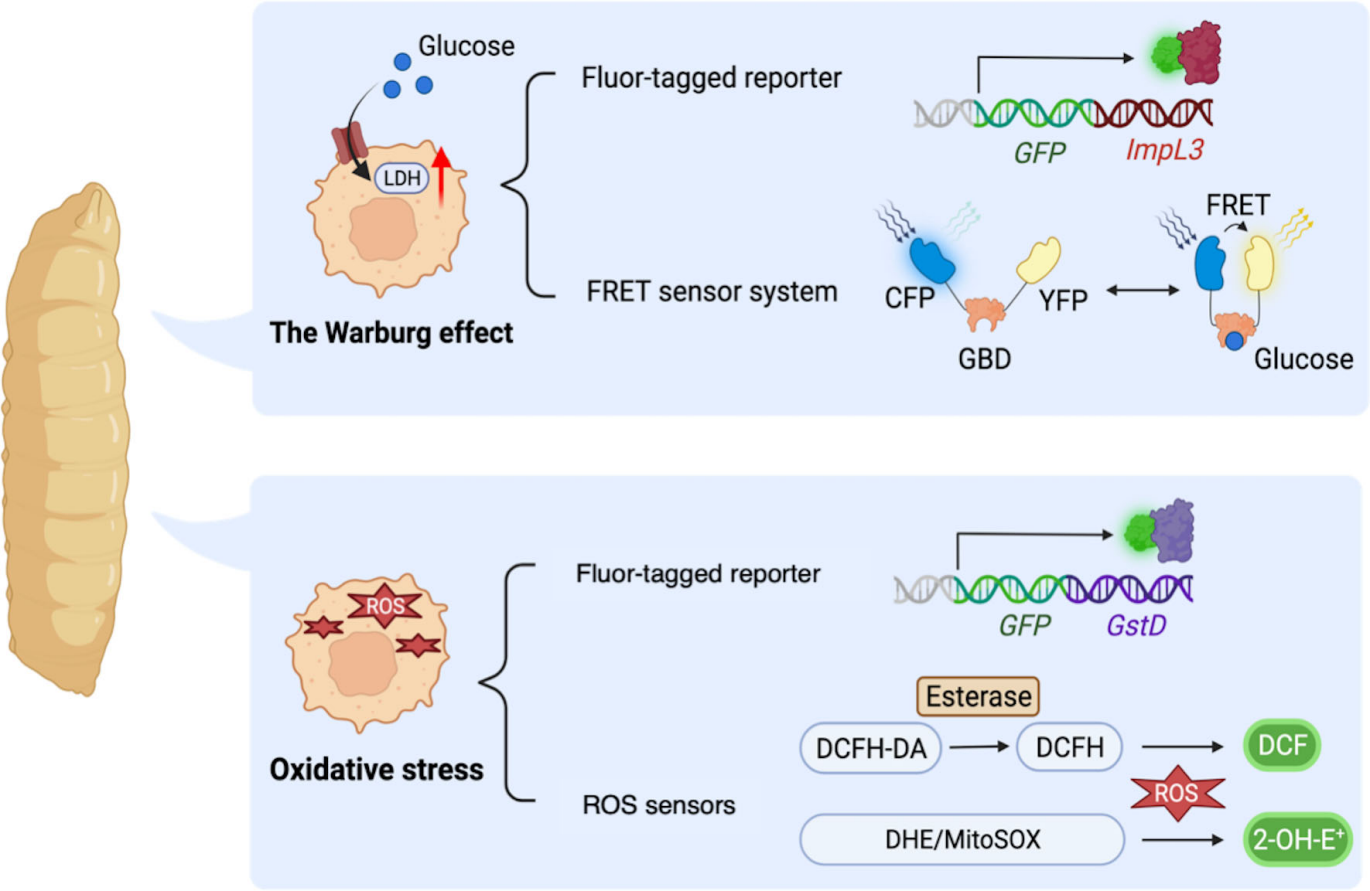
*Drosophila* methodologies to monitor metabolic alterations in a whole-body manner. *ImpL3*-*GFP*: a GFP-based enhancer trap reporter strain which enables easy detection of endogenous *ImpL3* expression in fly tissues. Förster resonance energy transfer (FRET)-based glucose sensor: a reporter strain carrying a glucose-binding domain (GBD), cyan fluorescent protein (CFP), and yellow fluorescent protein (YFP). FRET sensor determines the intracellular glucose level upon binding of glucose to GBD, which in turn changes the GBD’s structure to increase the FRET efficiency (the ratio of YFP to CFP). *GstD*-*GFP*, a GFP-based endogenous *GstD* expression reporter strain to monitor oxidative stress response. DCFH-DA, 2′,7′-Dichlorofluorescin diacetate, a cell-permeable ester that can be hydrolyzed intracellularly by esterases to become DCFH which reacts with H_2_O_2_ and turns into highly fluorescent DCF. DHE, dihydroethidium which forms a highly fluorescent product 2-hydroxyethidium (2-OH-E^+^). MitoSOX, a DHE derivative with an additional triphenylphosphonium group to target mitochondria monitoring ROS within mitochondria.

Firstly, fly models for cancer genotypes have shown to shift their glucose metabolism toward the Warburg effect. A study demonstrated that activation of PDGF/VEGF-receptor Pvr in imaginal discs induced epithelial tumors with upregulated ImpL3 and enhanced glycolysis (126). Indeed, *Pvr* activation induced glucose metabolic changes through stabilization of Hifα [a fly ortholog of human Hypoxia-inducible factor-1α (HIF-1α)], which transcriptionally upregulated glycolytic enzymes including ImpL3 inducing glycolysis. In this study, the authors employed a GFP-based enhancer trap reporter strain *ImpL3*-*GFP* to visualize *ImpL3* transcription, which enabled easy detection of endogenous *ImpL3* expression in fly tissues (**Figure 3**) (136). Additionally, the authors found that multiple oncogenic pathways inhibited activation of pyruvate dehydrogenase (PDH) which converts pyruvate into acetyl-CoA and is essential for driving the TCA cycle and oxidative phosphorylation.

Another paper also revealed elevated aerobic glycolysis in flies with misexpression of *Drosophila* Homeodomain-interacting protein kinase (Hipk). These *dpp*>*Hipk* flies displayed tumor formation, loss of epithelial integrity, and an invasion-like phenotype in their wing discs (132). The authors identified that *Hipk* triggered upregulation of *dMyc* in these transformed cells. It was already reported that induction of *dMyc* increased expression of glycolytic genes such as *ImpL3* hence upregulated glucose consumption as revealed in *Drosophila* S2 cells (137). As for *Hipk*-induced fly transformed cells, a study identified that *Hipk* triggers robust expression of glycolytic genes especially *ImpL3* and *Phosphofructokinase 2* (*Pfk2*, a fly ortholog of human *PFKFB*). Pfk2 catalyzes the synthesis of fructose-2,6-bisphosphate to stimulate further steps in glycolysis (122). Intriguingly, ectopic expression of *dMyc* was sufficient to increase *Pfk2* expression, leading to further *dMyc* accumulation in fly wing discs. These results suggest a positive feedback loop between *dMyc* and aerobic glycolysis (132). In this study, the authors monitored glucose metabolism in fly tissues with Förster resonance energy transfer (FRET)-based glucose sensor composed of a glucose-binding domain (GBD) combined with cyan fluorescent protein (CFP) and yellow fluorescent protein (YFP) to determine the intracellular glucose level (**Figure 3**) (123, 138). Binding of GBD to glucose induces GBD’s structural changes to increase the FRET efficiency (the ratio of YFP to CFP). These papers show that flies exhibit Warburg-like metabolic changes resulting from multiple molecular mechanisms, which has led us to better understand how the Warburg effect contributes to cancer cell metabolism. Moreover, fly platforms are able to provide novel insights of relations between signaling pathways involved in cancer and its metabolism.

Regarding Notch signaling pathway which is one of the most commonly mutated genes in cancer, previous papers demonstrated that *Notch*-induced fly models for cancer genotypes harbor accelerated glycolysis. One study established fly models with ectopic expression of *Drosophila* Notch intracellular domain (Nicd) in *ptc*>*Nicd* flies (139). In this paper, the authors performed a chromatin immunoprecipitation (ChIP) assay with antibody against Suppressor of Hairless [Su(H)], a key transcription factor in Notch signaling (140). This assay revealed that Notch signaling transcriptionally regulated several effectors in glycolysis including *ImpL3*. In addition, the aforementioned study by Slaninova et al. showed that knockdown of genes associated with glucose metabolism suppressed tissue overgrowth that Notch induced (139). These data suggest that a shift of normal glucose metabolism toward the Warburg effect is essential to promote tumorigenesis.

Another study also identified a relationship between Notch signaling pathway and the Warburg effect. The authors demonstrated how the Warburg effect causes cell proliferation using eye disc as a model tissue (141). They first performed genetic modifier screening with RNAi fly strains (109 genes in total) against *Notch*-induced fly tumor models with overexpression (OE) of *Drosophila* Notch ligand Delta (Dl) in eye discs [*eyeless* (*ey*)>*DI* ^OE^]. This screening revealed that *COX7a* (a mitochondrial respiratory chain subunit Cytochrome C-oxidase subunit 7a) was a key enhancer of eye cell proliferation induced by Notch activation. Furthermore, they found that *COX7a* knockdown attenuated the mitochondrial electron transport chain (ETC), and this attenuation resulted in Warburg-like metabolic changes such as upregulation of *ImpL3* expression as well as the intracellular lactate level. Moreover, they identified that upregulation of *ImpL3* activity resulted in lactate accumulation, which reduced intracellular pH level and then contributed to proliferation of these transformed cells. Therefore, this paper demonstrated that the Warburg effect may not only be a feature of glucose metabolism specific to transformed cells but also be a key promoter of cell proliferation. These papers prove that utilizing fly genetics disclose associations between genes or signaling pathways and the Warburg effect, which has potential to understand the fundamental roles of glucose metabolism in cancer cells.

Other than focusing on upregulation of *ImpL3* under oncogenic stress, a previous study demonstrated that ImpL3 itself is attributed to promotion of tumor-like phenotype in flies (103). In this paper, they demonstrated that *ImpL3* cooperated with *EGFR* to induce neoplasia. Specifically, co-expression of *ImpL3* with *dEGFR* in *apterous* (*ap*)>*dEGFR*, *ImpL3* flies led to tumor-like phenotypes in wing discs accompanied with increased level of MMP1 and loss of cell polarity. Additionally, they used the same flies to show that HSD promoted *EGFR*-induced neoplasia in an *ImpL3*-dependent manner. Consistently, concomitant activation of *LDHA* and *EGFR* was associated with poor patient prognosis in breast cancer, sarcoma, and gliomas (103). Therefore, flies contribute to understanding the molecular basis of the Warburg effect as well as prognostic markers for cancer patients. It is interesting to speculate that these mechanisms provide links between cancers and high sugar conditions such as DM.

In summary, flies share regulators and processes in glucose metabolism with humans. Thus, flies are useful for elucidating the mechanisms of glucose metabolism and its relationship with tumorigenesis. What makes this possible includes various whole-body tools to monitor glucose metabolism such as *ImpL3*-*GFP* reporter and FRET systems.

### Redox metabolism

Reactive oxygen species (ROS) is a group of highly reactive and heterogeneous molecules, including superoxide anion (•O_2_^−^), hydrogen peroxide (H_2_O_2_), and hydroxyl radicals (•OH), which are reduced oxygen generated from electron-leakage in the electron transport chain (121, 142). In a normal cell, ROS homeostasis is well sustained by the balance between ROS production and numerous detoxification processes regulated by antioxidant enzymes (124). On the other hand, the story is quite different in cancer cells: oxidative stress caused by excessive amounts of ROS can lead to oxidative modification-induced damage in intracellular macromolecules, which accumulates over time and ultimately causes cell death (9).

Along with aerobic glycolysis shown in section 4.1 (the Warburg effect), cancer cells also undergo reprogramming of mitochondrial metabolism, which causes the loss of redox homeostasis mainly by excessive production of ROS (**Figure 2**) (143). Indeed, a study reported that almost all cancer cells exhibited elevated levels of endogenous ROS (144). As such, oxidative stress is a result of metabolic reprogramming and is also known to be an important factor in tumor progression. In other words, ROS is a double-edged sword for tumor progression depending on its concentration. Namely, mild elevation of ROS makes it a second messenger necessary for many aspects of tumor development and progression (145). For example, low concentration of ROS stimulated proliferation of cultured human cells of various cancers such as breast and ovarian cancers by directly inhibiting GDP/GTP exchange within RAS hence activating RAS-ERK1/2 signaling through oxidative modification (145, 146). In contrast, high concentration of ROS is toxic to cancer cells by directly inducing cancer cell death through senescence, apoptosis, and ferroptosis (144, 145). Abundant ROS also inhibits cancer progression by sensitizing drug-resistant cancer cells (144). In addition, previous studies have revealed that exogenous H_2_O_2_ triggered cancer cell death with a high basal level of ROS in the pancreas and brain (147–149). In pancreatic cancer cells in particular, intracellular elevation of antioxidants derived from increased activity of antioxidant proteins is a prerequisite for the occurrence of tumor hallmarks including cell proliferation and metastasis (147). However, the complexity of ROS in cancers remains to be an important question to be addressed.

Therefore, researchers tried different ways to observe metabolic impacts of ROS. Increasing number of fly studies indicated that *Drosophila* is a well-suited model organism to study metabolic reprogramming in the redox process. This is because over 90% of ROS is derived from energy metabolism in mitochondria fly regulators of which are highly conserved with humans (150, 151). In fact, the utilization of flies as a whole-body organism to reveal the redox process in diseases including cancers has increased over the past few years. Previous studies of aging (152), obesity (93), diabetic retinopathy (153, 154), and neurodevelopmental diseases (155) have successfully modeled in flies the oxidative stress in human diseases.

On the other hand, emerging diversity of methodologies in flies to quantify the ROS amount also offers advantages to make *Drosophila* a suitable model for understanding redox metabolism. As mentioned above, metabolites and metabolic pathways in flies and mammals are highly conserved. Therefore, many established tools for studying metabolic changes in mammals can be directly applied to fly studies (156). So far, there exist a variety of tools established to directly measure intracellular ROS. For example, dihydroethidium (DHE) is one of the most frequently used dyes which fluoresces upon oxidation by superoxide (157). In addition, MitoSOX is used to distinguish the sources of ROS as a modified version of DHE with a mitochondrion-targeting group to observe ROS that mitochondria generate (156, 158). Another widely used fluorogenic probe for oxidative stress in mammals is 2′,7′-Dichlorofluorescin diacetate (DCFH-DA) probe, which also proved efficient in flies (153, 155, 159, 160). Furthermore, as a useful genetic tool in flies, the reporter gene *GstD* can easily quantify intracellular ROS levels. The fly *GstD* is an oxidative stress response gene encoding for glutathione S-transferase (161). Since expression of *GstD* is positively correlated with intracellular oxidative stress, transgenic flies carrying *GstD*-*GFP* are developed to conveniently evaluate intracellular ROS levels in disease models (**Figure 3**) (71, 162).

For the past several years, an increasing number of fly studies unraveled ROS-related redox metabolic reprogramming in cancer cells with various genotypes. Indeed, these studies encompassed a wide range of signaling pathways (**Table 2**). For example, a group established a glycolytic tumor model in flies by activating the oncogenic *Pvr* (126). In this model, they found that excess ROS produced in transformed cells functioned as a feedback signal to consolidate glycolytic metabolic reprogramming. Moreover, *dMyc* induction in wing disc epithelium increased ROS substantially, which may transduce pre-cancerization effect by dMyc to adjacent tissues (130). In addition, *Hipk*-overexpression induced ROS in transformed cells by inhibiting mitochondrial energetics, which exacerbated tumors by potentiating JNK and its downstream MMP1 (132). Strikingly, almost all tumorigenic mutations tested thus far produced extra ROS in transformed cells regardless of tissue types in *Drosophila*. Exceptions include *ras* ^G12V^ which suppressed ROS production (70, 129), and this outcome is in accordance with ROS detoxification *via* the RAS-RAF-NRF2 pathway (10).

Moreover, loss of cell polarity caused by elevated ROS is a well-studied phenotype related to redox metabolism (124). The discovery of important genes regulating cell polarity in *Drosophila* makes it possible to establish fly cancer models with loss of such regulators to unravel metabolic reprogramming in tumor cells that had lost their polarity (125, 127, 129, 163). For example, *discs-large* (*dlg)* and *scrib* are two important genes to maintain cell polarity in flies (164). Loss of *dlg* in epithelial cells of larval wing discs causes overgrowth due to loss of cell polarity. In addition, DHE staining of wing discs demonstrated higher superoxide levels in those transformed cells (125). Likewise, *ras* ^G12V^,*scrib* ^−/−^ flies mimic loss of cell polarity in the context of cellular transformation (129) (127, 163). Though transformed cells carrying *ras* ^G12V^ or *scrib* ^−/−^ alone did not show intracellular oxidative stress, transformed cells with the concurrence of *ras* ^G12V^ and *scrib* ^−/−^ produced ROS by structurally damaged mitochondria (129). Furthermore, another group investigated the role of ROS in signaling pathways of transformed cells and demonstrated that *ras* ^G12V^-activated caspases increased intra- and extra-cellular ROS rather than inducing apoptosis in transformed cells. These results indicated that ROS promoted a caspase-triggered amplification loop and promoted tumor progression (127).

Besides these ROS alterations under loss of cell polarity, elevated ROS also showed up in transformed cells in flies modeling brain cancer genotypes (128, 131, 135). A study established a tumor model *da*>*brat* ^RNAi^ to show elevated levels of ROS and chromosomal instability (CIN) by depleting the *brain tumor* (*brat*) gene in the brain of third instar larvae using the *daughterless* (*da*) driver which is active in fly neurons. Moreover, extracellular antioxidants blocked overgrowth of *brat*-deleted tumors, showing the essential role of ROS elevation in CIN-dependent tumorigenesis. Hence, accumulated ROS can be a vulnerability for CIN-dependent tumors that can be targeted by metabolic intervention (128). However, whether accumulated ROS promotes tumor cell proliferation is currently inconclusive. In another study focusing on brain cancers, authors developed *brat*-deleted tumors by using *Pointed* (*pnt*) and *Asense* (*ase*) drivers targeting neuroblasts in larval brains of *pnt*>*brat* ^RNAi^ and *ase*>*brat* ^RNAi^ flies. Their results showed that scavenging ROS by antioxidant treatment did not affect the tumor progression, though the tumors contained significantly elevated ROS than normal larval brain. Instead of oxidative stress by ROS, reprogrammed redox homeostasis of NAD^+^/NADH is primarily required for *brat*-deleted tumors to become immortalized (131). Therefore, the role of ROS elevation led by *brat*-deletion in fly neuroblasts still remains unraveled needing further investigation. On the other hand, a study induced *Notch* overexpression using the *1407*-GAL4 driver to develop proliferative transformed cells in the brain of *1407>N* ^OE^ flies. These transformed cells exhibited elevated ROS production triggered by Notch-RET-signaling to contribute to Notch-induced neoplastic transformation (92, 135).

With the established ISC tumors that *esg*-GAL4 drives in flies, there have been multiple studies on the role of ROS under various physiological and pathological conditions (133). For example, a study identified an intrinsic homeostatic range of ROS in ISCs, indicating that the intracellular redox level is a critical determinant of cancer cell fate (71). In this study, tumor-like ISCs induced by depleting *Notch* in *esg*>*N* ^RNAi^ flies and extracellular matrix (ECM)-deprived ISCs induced by depleting *β*-*integrin* (*mys*) in *esg*>*mys* ^RNAi^ flies exhibited proliferative phenotype under a moderate increase of ROS. On the other hand, ISCs with both *N*- and *mys*-reduction (*esg*>*N* ^RNAi^,*mys* ^RNAi^) displayed metastatic phenotypes accompanied by even higher ROS levels with cytotoxic oxidative stress (71). Additionally, another paper focused on tumor microenvironment regulating ROS. The authors utilized *esg*>*Raf* ^act^ flies modeling benign gut tumors by targeting a constitutively active form of *Raf* to adult intestines (133). They confirmed that autophagy in cells around neoplasia was induced downstream of elevated ROS and activated JNK signaling in tumor cells. Transformed cells had significantly increased ROS, while ROS elevation was mild in their neighboring cells. Intriguingly, sparing expression of the antioxidase catalase gene in transformed cells efficiently blocked autophagy in surrounding cells and inhibited tumor proliferation (149).

Beside revealing the role of intracellular ROS in tumor cells, there are studies using *Drosophila* to identify the relationship between tumor-derived ROS and cardiac dysfunction. For example, fly models with *yki*-overexpression had a systemic increase in ROS, which resulted in compromised cardiac function (134).

To summarize, flies share conserved redox metabolism pathways with humans, and previous studies have provided novel insights into cancer redox metabolism using fly models of cancer genotypes. Emerging diversity of methodologies in flies to evaluate redox metabolism in transformed cells provides flies with potential in elucidating the mechanisms of cancer redox metabolism and its relationship with carcinogenesis.

## Conclusion

In this review, we highlighted *Drosophila* studies on cancer demonstrating the cancer mechanisms and unique metabolism. Recently, we have access to flies produced to carry a variety of cancer driver mutations. These flies have surpassed the usage in studying cancer signaling pathways and contributed to drug discovery in a high-throughput manner. Furthermore, the broad application of flies in metabolic disease research has demonstrated that the high similarity between fly and human metabolism allows for the reproduction in flies of characteristic metabolic changes in human diseases to elucidate their mechanisms and their impact on concurring diseases. Based on this idea, many studies have come up with new insights into cancer metabolism by analyzing fly models for various cancer genotypes carrying markers. Therefore, we expect that *Drosophila* keeps playing a significant role in our future exploration of the nature of cancer as a systemic disease and in providing candidate targets for novel therapeutics against notorious cancers.

## Author contributions

HJ, TK, HH: Writing—Original draft, Writing—Review and editing. MS, RY: Conceptualization, Writing—Review and editing, Project administration, Funding acquisition. All authors contributed to the article and approved the submitted version.

## Funding

The Sonoshita lab is supported by grants from the MEXT/JSPS KAKENHI (Grant Number 19H05412, 19K22478, 20H03524, 20K07558, and 21K14762), AMED (Grant Number JP20ck0106548, JP20cm0106273, and 22ak0101163), JST START (Grant Number ST211005JS), the JSPS COI Next Generation Researchers Collaborative Research Fund (Grant Number R03W01), Suzuken Memorial Foundation, Extramural Collaborative Research Grant of Cancer Research Institute, Kanazawa University, Ichiro Azuma Foundation, G-7 Scholarship Foundation, The Translational Research program; Strategic PRomotion for practical application of INnovative medical Technology (TR-SPRINT), MSD Life Science Foundation, SGH Foundation, Foundation for Promotion of Cancer Research in Japan, Project Mirai Cancer Research Grants, The Ichiro Kanehara Foundation, The Princess Takamatsu Cancer Research Fund, The Mochida Memorial Foundation for Medical and Pharmaceutical Research, The Suhara Memorial Foundation, The Tokyo Biochemical Research Foundation, Japan Foundation for Applied Enzymology, Takeda Science Foundation, The Akiyama Life Science Foundation, All Japan Coffee Association, and The Pharmacological Research Foundation, Tokyo.

## Acknowledgments

We thank all members of the Sonoshita lab for their support in this work. HJ and HH were supported by JST SPRING Scholarship. TK was supported by the Iwadare Foundation Scholarship. The Sonoshita laboratory was supported in part by the Joint Usage/Research Center for Genetic Medicine, Hokkaido University and the Photo-excitonix Project in Hokkaido University. Figures in this review are created with BioRender.com.

## Conflict of interest

The authors declare that the research was conducted in the absence of any commercial or financial relationships that could be construed as a potential conflict of interest.

## Publisher's note

All claims expressed in this article are solely those of the authors and do not necessarily represent those of their affiliated organizations, or those of the publisher, the editors and the reviewers. Any product that may be evaluated in this article, or claim that may be made by its manufacturer, is not guaranteed or endorsed by the publisher.
